# Acute eosinophilic pneumonia accompanied with COVID‐19: a case report

**DOI:** 10.1002/rcr2.683

**Published:** 2020-11-16

**Authors:** Koutaro Murao, Atsushi Saito, Koji Kuronuma, Yoshihiro Fujiya, Satoshi Takahashi, Hirofumi Chiba

**Affiliations:** ^1^ Department of Respiratory Medicine and Allergology Sapporo Medical University School of Medicine Sapporo Japan; ^2^ Department of Infection Control and Laboratory Medicine Sapporo Medical University School of Medicine Sapporo Japan

**Keywords:** Acute eosinophilic pneumonia, COVID‐19, SARS‐CoV‐2

## Abstract

We report a case of acute eosinophilic pneumonia (AEP) triggered by coronavirus disease 2019 (COVID‐19) infection. A 77‐year‐old man experienced left‐sided chest pain and shortness of breath. Reverse transcription‐polymerase chain reaction (RT‐PCR) for severe acute respiratory syndrome coronavirus‐2 (SARS‐CoV‐2) revealed a positive result, and he was treated with favipiravir, ciclesonide, and lascufloxacin, but he showed poor improvement. On the other hand, computed tomography (CT) images were atypical for COVID‐19 infection, and the elevation of eosinophil was found in blood and the fluid obtained by bronchoscopy. So, we clinically diagnosed this case as AEP. Administration of prednisolone dramatically improved the patient's clinical condition and chest radiograph findings, which were consistent with the clinical course of AEP. This case suggests the importance of considering the complications of AEP when treating patients with COVID‐19 infection.

## Graphical abstract

We report a case of acute eosinophilic pneumonia (AEP) triggered by coronavirus disease 2019 (COVID‐19) infection. This case suggests the importance of considering the complications of AEP when treating patients with COVID‐19 infection.

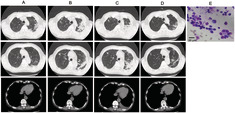

## Introduction

Acute eosinophilic pneumonia (AEP) is an acute rare lung disease characterized by respiratory failure and consolidation and diffuse ground‐glass opacities on chest computed tomography (CT). AEP has been described in association with drugs, smoking, and infectious diseases, including viral infections, or otherwise as idiopathic. In this report, we describe the first case of AEP accompanied with coronavirus disease 2019 (COVID‐19), which is caused by the severe acute respiratory syndrome coronavirus‐2 (SARS‐CoV‐2).

## Case Report

**Figure 1:**
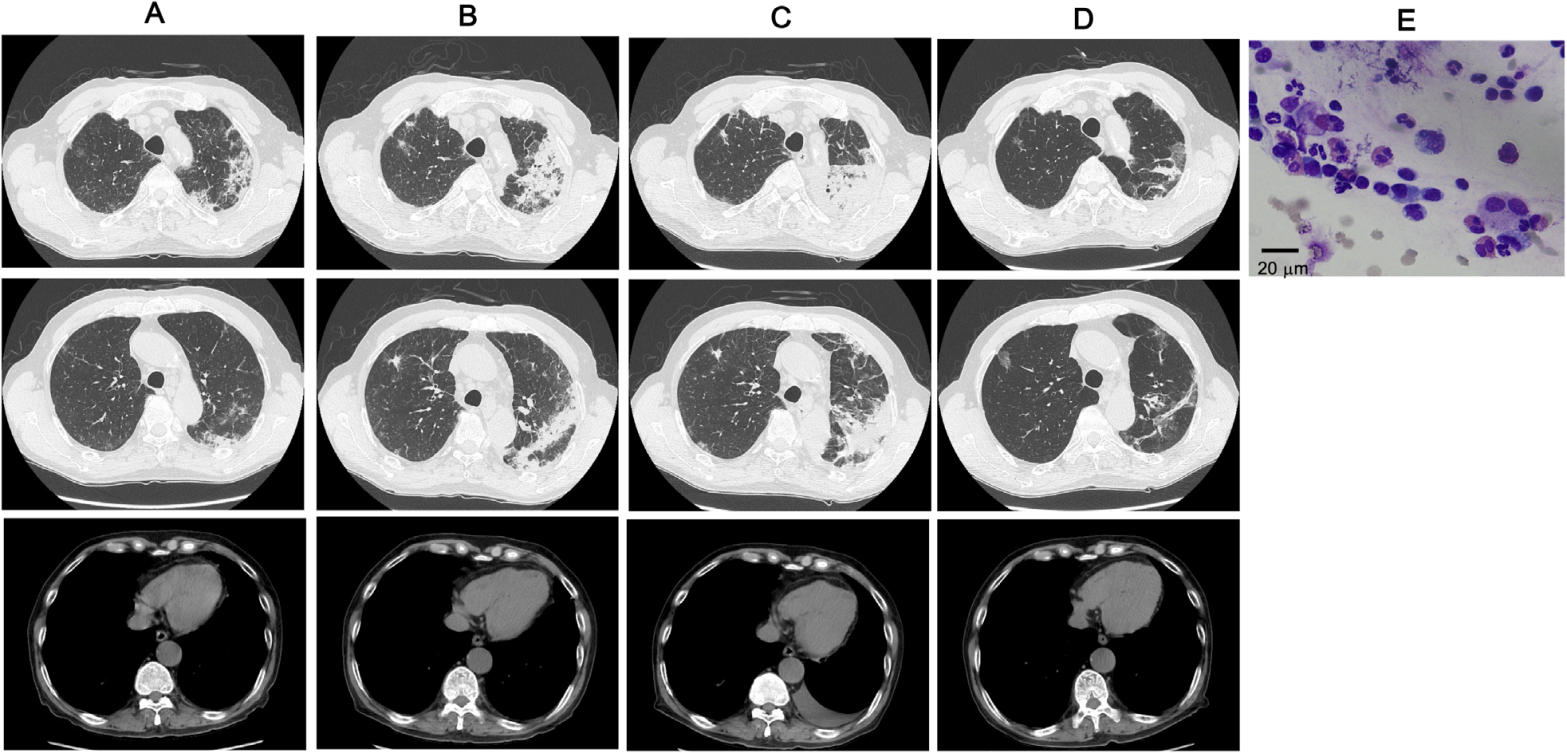
Serial chest computed tomography (CT) images of the patient on (A) hospital day 1, (B) hospital day 11, (C) hospital day 21, and (D) hospital day 46, and the elevation of eosinophil in the lung. (A, B) Bilateral upper and middle areas of ground‐glass opacities and consolidations at the subpleural and peribronchial regions. (C) Pleural effusions were observed in the left side. (D) Post‐treatment with prednisolone (PSL). (E) The fluid obtained by bronchoscopy were cytospun onto glass slides and stained with Giemsa's staining solution. Representative image is shown. Bold scale bar, 20 μm.

A 77‐year‐old man with a history of bronchial asthma experienced left‐sided chest pain and shortness of breath. He was subsequently referred to our hospital with bilateral pneumonia on chest radiograph and a positive reverse transcription‐polymerase chain reaction (RT‐PCR) result for SARS‐CoV‐2. On examination, his body temperature was 37.7°C, heart rate was 105 beats/min, and oxygen saturation was 98% (room air). Blood examination showed the following results: white blood cells (WBC) 8.4 × 10^3^/μL (neutrophils: 72.3%, eosinophils: 12.1%), haemoglobin (Hb) 15.2 g/dL, platelets 30 × 10^4^/μL, Na 138 mEq/L, K 4.4 mEq/L, Cl 105 mEq/L, C‐reactive protein (CRP) 5.93 mg/dL, blood urea nitrogen (BUN) 23 mg/dL, creatinine (Cre) 0.80 mg/dL, aspartate aminotransferase (AST) 15 IU/L, alanine aminotransferase (ALT) 11 IU/L, and lactate dehydrogenase (LDH) 149 U/L. These data indicated a typical finding in the early stages of COVID‐19 infection, except that there was a slight elevation in the eosinophil count on admission. Chest CT showed ground‐glass attenuations (GGA) and consolidation (Fig. 1).

**Figure 2:**
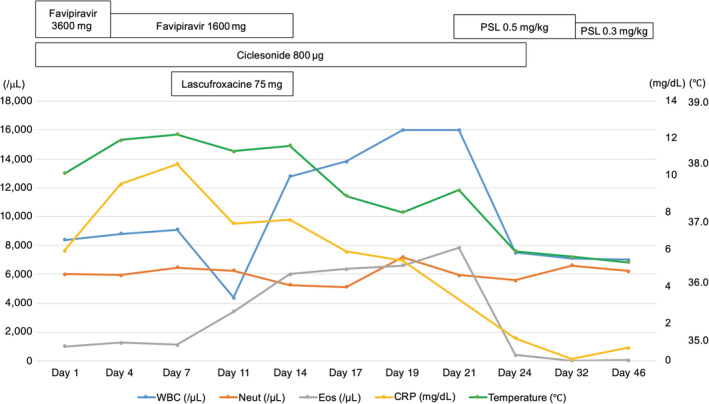
Clinical course of the patient. The number of eosinophils gradually increased for the initial 21 days. After the start of prednisolone (PSL) administration, the number of white blood cells and eosinophils promptly decreased.

After hospitalization, ciclesonide (800 μg/day) and favipiravir, which is one of the antiviral drug, were started with the consent of the patient, as he was relatively old and had respiratory complications. The dose of favipiravir was 3600 mg (1800 mg twice daily (bd)) on day 1, followed by 1600 mg (800 mg bd) from day 2. As shown in Figure 2, his CRP levels gradually increased, and the GGA shifted to consolidation (Fig. 1). Chest CT images appeared to be atypical for COVID‐19‐related pneumonia with suspected involvement of bacterial pneumonia, and hence the patient was treated with lascufloxacin (75 mg/day). However, a mild fever continued after that accompanied by a gradual increase in the number of eosinophils (Fig. 2). As we considered the possibility of drug‐induced eosinophilia due to the side effects of favipiravir or lascufloxacin, we discontinued the administration of both drugs. However, blood examination revealed worsened eosinophilia, and the CT images disclosed the exacerbation of consolidation and the appearance of pleural effusion.

As we could not relate the deteriorated radiological changes and the eosinophilia to COVID‐19‐associated pneumonia alone and increased eosinophils were found in fluid obtained by a bronchoscopy (Fig. 1E), we clinically diagnosed this case as AEP accompanied by COVID‐19, and the patient was started on a course of prednisolone (PSL) (0.5 mg/kg). Subsequently, his eosinophil count decreased markedly and the chest CT showed reduction and disappearance of the GGA, consolidation, and the left pleural effusion. The patient was discharged home with instructions for a prolonged PSL taper. Approximately one month later, his peripheral eosinophil count decreased to normal levels and the chest CT showed no abnormal findings.

## Discussion

Several COVID‐related complications have been reported till date, and it has been indicated that primarily systemic inflammation and pulmonary complications can lead to significant morbidity and mortality. On the other hand, AEP is one of the pulmonary complications that has not yet been reported, as observed in the present case. The clinical course in this case was slightly different from that of general COVID‐19 cases, suggesting the possibility of AEP. In general, AEP develops with the symptoms of fever, dry cough, dyspnoea, chest pain, and elevation of CRP levels in the blood test. In our case, we clinically diagnosed as AEP based on his clinical symptoms, blood test results, increased eosinophils in fluid obtained by a bronchoscopy, and the improved response to PSL. Although drug‐induced eosinophilic pneumonia (EP) caused due to favipiravir or lascufloxacin should be considered in the differential diagnosis, its possibility can be excluded as there was no radiological improvement and reduction of eosinophil count after stopping the medication. Considering these findings together, we concluded that this case was AEP triggered by COVID‐19 infection.

It is well known that EP is caused by viral infection [1]. Eosinophils may contribute to the lung pathology induced during COVID‐19 infection [2]. However, it is still unknown whether eosinophils have a protective or exacerbating role during SARS‐CoV‐2 infection. Interestingly, Zhang et al. reported that more than half of the patients admitted with COVID‐19 infection (53%) had eosinopenia on the day of hospitalization [3]. Similarly, Du et al. reviewed the medical records of 85 fatal cases of COVID‐19 and observed that 81% of patients had absolute eosinophil counts below the normal range at the time of admission [4].

Importantly, no eosinophil enrichment into the pulmonary tissue has been observed in samples from patients with COVID‐19 infection during the early stages of disease or in post‐mortem analyses [5]. In our case, eosinophilia was induced by SARS‐CoV‐2 infection and influenced the clinical course.

Corticosteroids are not routinely recommended for COVID‐19 treatment and might exacerbate COVID‐19‐associated lung injury. Furthermore, we hypothesize that the use of corticosteroids is associated with sustained detection of SARS‐CoV‐2. Nevertheless, it is necessary to consider steroid treatment in patients with COVID‐19 infection when complicated with AEP or cryptogenic organizing pneumonia.

This case suggests that the comorbidity of AEP should also be considered when treating patients with COVID‐19 infection.

### Disclosure Statement

Appropriate written informed consent was obtained for publication of this case report and accompanying images.

## References

[1] Larranaga JM , Marcos PJ , Pombo F , et al. 2016 Acute eosinophilic pneumonia as a complication of influenza A (H1N1) pulmonary infection. Sarcoidosis Vasc. Diffuse Lung Dis. 33:95–97. 27055842

[2] Lindsley AW , Schwartz JT , and Rothenberg ME . 2020 Eosinophil responses during COVID‐19 infections and coronavirus vaccination. J. Allergy Clin. Immunol. 146:1–7. 32344056 10.1016/j.jaci.2020.04.021 PMC7194727

[3] Zhang JJ , Dong X , Cao YY , et al. 2020 Clinical characteristics of 140 patients infected with SARS‐CoV‐2 in Wuhan, China. Allergy 75:1730–1741. 32077115 10.1111/all.14238

[4] Du Y , Tu L , Zhu P , et al. 2020 Clinical features of 85 fatal cases of COVID‐19 from Wuhan. A retrospective observational study. Am. J. Respir. Crit. Care Med. 201:1372–1379. 32242738 10.1164/rccm.202003-0543OC PMC7258652

[5] Barton LM , Duval EJ , Stroberg E , et al. 2020 COVID‐19 autopsies, Oklahoma, USA. Am. J. Clin. Pathol. 153:725–733. 32275742 10.1093/ajcp/aqaa062 PMC7184436

